# Psychological insulin resistance among patients with diabetes mellitus in Botswana

**DOI:** 10.4102/phcfm.v17i1.4752

**Published:** 2025-04-22

**Authors:** Engemadzo Bitsang, Billy M. Tsima, Keneilwe Motlhatlhedi

**Affiliations:** 1 Ministry of Health, Gaborone, Botswana; 2 Department of Family Medicine and Public Health, Faculty of Medicine, University of Botswana, Gaborone, Botswana

**Keywords:** diabetes mellitus, psychological insulin resistance, insulin therapy, ITAS scale, Botswana

## Abstract

**Background:**

Timely initiation of insulin therapy is vital in patients with type 2 diabetes mellitus. However, insulin initiation may be delayed because of psychological insulin resistance (PIR).

**Aim:**

This study aims to determine the prevalence of PIR and factors that contribute to PIR in adults with type 2 diabetes mellitus at a diabetes clinic in Botswana.

**Setting:**

The study was conducted in a diabetes clinic, one of the two large centres in the country that manage diabetes mellitus. It is situated on the south eastern part of Botswana.

**Methods:**

This was a cross-sectional study among patients with type 2 diabetes mellitus. We included participants aged between 18 and 80 years old and diagnosed with type 2 diabetes mellitus for more than 1 year. Patients’ attitudes towards insulin therapy were assessed using the Insulin Treatment Appraisal Scale (ITAS).

**Results:**

The prevalence of PIR was 82.9% (95% confidence interval [CI]: 78.0 – 87.8) out of the 228 respondents. Factors significantly associated with PIR were gender (odds ratio [OR] adjusted 0.44; 95% confidence interval 0.211 – 0.921; *p* = 0.029) and treatment type (OR adjusted 1.58; 95% confidence interval 1.067 – 2.341; *p* = 0.023).

**Conclusion:**

Significant factors associated with psychological insulin resistance were patient and health system-related. It is therefore pivotal to improve patient and healthcare provider communication and to ensure that patient education is thoroughly performed at the diagnosis of disease.

**Contribution:**

This study highlights the importance of patient education at diagnosis and during treatment continuum of diabetes mellitus.

## Introduction

Globally, more than 500 million people live with diabetes and over 6 million died from diabetes-related complications in the year 2021. Furthermore, the number of people living with diabetes is projected to increase by over 90% in sub-Saharan Africa by 2030.^1^

Diabetes mellitus is associated with significant complications, which profoundly affect patients’ and their families’ quality of life. Vascular complications of diabetes are well recognised and occur as a result of disease progression. These include microvascular complications such as retinopathy, neuropathy and nephropathy as well as macrovascular complications such as cardiovascular diseases, stroke and amputation.^2^ Good control of blood glucose is important in order to delay onset or slow progression of diabetes-related complications.

Insulin treatment is considered one of the most effective diabetes treatment interventions. It is no longer considered an option of last resort in type 2 diabetes. It is increasingly becoming more valued because of its ability to promote appropriate levels of glycaemic control, prevent complications and at the same time have no significant effect on the patient’s quality of life.^3,4^

Despite the proven efficacy of insulin compared to oral hypoglycaemic agents, patients are reluctant to be started on insulin therapy.^5^ Moreover, some physicians are reluctant to start patients on insulin as they worry about hypoglycaemia, monitoring of blood glucose levels, weight gain with insulin and also believe its initiation signals the last resort in the continuum of medical management of diabetes mellitus.^6^

Psychological insulin resistance (PIR) is a broad concept that covers multiple factors involved in the refusal of insulin as a treatment option for management of diabetes. It refers to psychological barriers to initiation and persistence with insulin therapy.^7^ Factors described include: emotional states (needle anxiety, fear of hypoglycaemia, weight gain), cognitive (a lack of knowledge, distorted beliefs about insulin treatment), attitudes and behaviours of healthcare providers as well as the influences from the social and cultural environment.^7^

Psychological insulin resistance has drawn increasing attention to diabetes patients’ well-being, as well as to the overall cost of diabetes management because of its multiple implications.^8^ Assisting healthcare professionals to better understand PIR from the patient’s perspective is likely to result in improved treatment outcomes. By tailoring treatments to patients’ PIR status, clinicians may be able to help their patients better and begin insulin treatment timely and improve compliance, thus facilitating target glycaemic control achievements and improved clinical outcomes.

The prevalence of diabetes is rising in low and middle income countries. In Botswana, it is estimated that between 5% and 7% of the adult population has diabetes mellitus.^1^ This rise in cases of diabetes mellitus translates to a rise in care demands and eventually increased diabetes-related morbidity and mortality if effective management strategies are not employed. There is a scarcity of information about PIR in Botswana. Determining the prevalence and factors associated with PIR in adults with type 2 diabetes mellitus may help improve patient care and decrease diabetes mellitus-related complications in Botswana.

## Research methods and design

### Study design

This was a cross-sectional study.

### Study period and setting

Data collection was carried out between February and March 2020 at a diabetes clinic, one of two diabetes centres of excellence in Botswana. The clinic is an extension of a referral hospital situated in a city, south eastern part of Botswana. The city holds a population of approximately 200 000. The clinic serves a diverse range of patients as it also receives referrals from surrounding towns and villages. It offers various services to an estimated 3000 diabetic patient base. Services include physician consultations, health education, eye and foot screening as well as issuing of medicines. On average approximately 2000 diabetic patients visit the clinic monthly to access these services and an average of 1400 of these patient visits are physician consultations.

### Study population

The study was conducted among adults with type 2 diabetes mellitus, followed or attended at the diabetes clinic. We included patients 18–80 years old diagnosed with type 2 diabetes for more than 1 year at the time of the study. Patients with type 1 diabetes mellitus, pregnant women, patients with acute psychotic illness, dementia or critically ill patients were excluded.

### Sample size and sampling

The sample size for the study was determined using Cochran’s formula assuming a prevalence of PIR of 82.6% based on a study carried out in Kenya.^9,10^ The estimated minimum sample size required to determine the prevalence of PIR was 220.

The estimated sample size was inflated by 10% to account for non-response. The final sample size was therefore 244 patients with type 2 diabetes. The participants were selected by convenience (we approached any patient in the clinic seeking care at the time of the study, informed them of the study and if they agreed to take part and met the inclusion criteria, they were enrolled in the study) until the target number was reached. Stratification was used while sampling so that both patients on insulin therapy and those who were not would be relatively balanced (if we thought we were collecting more data from insulin naïve patients, we would stop and collect from those on insulin therapy).

### Piloting

Prior to the commencement of the study, the data collection tool was pretested and the resultant data were analysed to inform any need for correction and/or modifications of the tool. The data collection tool was pretested on 25 patients, 10% of the estimated sample size. Piloting was performed in the same setting as the main study. However, the 25 patients who took part in the pilot study were not included in the main study. The pilot study participants’ files were tagged to indicate their participation in the pilot study and exclusion from the main study. At the end no changes were made on the data collection tool after piloting. Data collection for the main study was conducted a month after the pilot.

### Data collection

Data collection was carried out using an investigator administered questionnaire to capture socio-demographics and clinical characteristics of participants, as well as participants’ responses to the insulin treatment appraisal scale (ITAS). The ITAS scale is a validated standard tool, which has been used in several studies across the globe to examine insulin-related attitudes and was created to quickly measure negative and positive beliefs regarding insulin.^10,11,12^

Prior to using the ITAS for the study, it was reviewed by relevant stakeholders (2 endocrinologists, 3 family physicians, a dietician, and several nurses) at the diabetes clinic. Few changes were made, including the following:

- Three additional questions were included to supplement questions in the ITAS scale. These additional questions were considered important to understand the participants’ attitudes towards use of insulin in the context of Botswana. However, these questions were not integrated into the original ITAS scale and were analysed separately from the original ITAS items.
- The words ‘blood glucose’ in the English version were changed to ‘blood sugar’ for alignment with language commonly used in the setting of our study.

The questionnaire was translated from English to Setswana and back translated to English. Forward translation was performed by an independent bilingual (English/Setswana) translator and back translation was carried out by a different translator who was blinded to the original English version. No significant differences between the two versions were identified.

Two interviewers (research assistants) trained by the authors on the study procedures administered the questionnaire in local vernacular language (Setswana) or English depending on the participants’ preference. Participants’ medical records were reviewed to verify diagnosis, treatment prescribed and laboratory results. The questionnaire took at least 15–20 min to complete.

### Statistical analysis

Data were entered on Microsoft Excel, checked for missing variables, consistency, completeness, exported to and analysed using IBM Statistical Package for the Social Sciences (SPSS) version 25.0. Continuous variables such as age, duration of diabetes diagnosis and glycated haemoglobin (HbAlc) levels were described in terms of means and standard deviations. Where the continuous data were skewed, median (and interquartile range) was used. Categorical data, for example, gender, marital status, education, income, type of treatment, family history of diabetes mellitus were presented as proportions or frequencies.

The ITAS instrument is a 20-point (four positive and 16 negative statements) tool that provides an appraisal of the patient on insulin therapy. Each statement has a 5-point Likert scale of responses. Insulin treatment appraisal scale scores range from 20 to 100, where lower scores of 40 and below represent more positive attitudes and beliefs about insulin therapy and scores above 40 represent more negative attitudes. The four positive statements were reverse scored before obtaining the total score as recommended by the scale developers.^13^ The foregoing interpretation was then applied to the study participants’ responses to obtain the summation score for each participant. Patients were then categorised into those with predominantly positive (scores below 40) or negative attitudes (scores above 40).^13^ Responses to each of the ITAS statements were also analysed and grouped into the five domains of PIR captured in the ITAS instrument namely: perceived personal blame, fear, self - pity or social stigma, perceived loss of one’s ability to control and dependence.

Means of variables with normally distributed continuous data were compared using the student’s *t*-test. The Chi-squared test was used to compare categorical data stratified by attitude (positive vs. negative). Univariable logistic regression modelling was performed to explore the relationship between the outcome (PIR) and selected exposure variables (socio-demographics and clinical factors). A multivariable logistic regression model was fit following the univariable logistic regression modelling analyses. Variables were considered for inclusion into the multivariable logistic regression model if they were significant (*P* < 0.25) in univariable logistic regression modelling analyses. A *p*-value of less than 0.05 was considered statistically significant in the final multivariable logistic regression model.

### Ethical considerations

Ethical clearance to conduct this study was obtained from the University of Botswana Institutional Review Board (IRB) (No. UBR/RES/IRB/BIO/GRAD/105) and the Ministry of Health and Wellness, Health Research and Development Division (No. HPDME-13/18/1). Further approval was sought from the study site management team.

Written informed consent was acquired from patients prior to participating in the study. No personal or hospital identification number was captured in the data collection tool. Data were kept in a password-protected personal computer at all times.

## Results

Of the 244 eligible patients approached to take part in the study, 228 agreed to participate, giving a response rate of 93.4%. The prevalence of PIR was 82.9% (95% confidence interval [CI]: 78.0–87.8). The participants’ sociodemographics and clinical characteristics stratified by PIR are shown in Table 1. The majority of participants were female (59.6%), almost half were married (47.4%), a third had primary school education (32%) and 36.8% had no income. Gender and treatment options distributions differed significantly according to PIR status (Table 1).

**TABLE 1:** Participants’ socio-demographic and clinical characteristics stratified by psychological insulin resistance status.

Variable	Total		No PIR (ITAS < 40)		PIR (ITAS > 40)		*P*
	*n*	%	*n*	%	*n*	%	
**Age (years)**							
< 40	21	9.2	3	14.3	18	85.7	-
40–49	33	14.5	7	21.2	26	78.8	0.775
50–59	71	31.1	12	21.2	59	83.1	-
60–69	66	28.9	13	19.7	53	80.3	-
> 70	37	16.2	4	10.8	33	89.2	-
**Gender**							
Male	92	40.4	23	25.0	69	75.0	0.009**
Female	136	59.6	16	11.8	120	88.0	-
**Marital status**							
Single	83	36.4	13	15.7	70	84.3	-
Married	108	47.4	21	19.4	87	80.6	0.963
Separated	2	0.9	0	0.0	2	100.0	-
Divorced	6	2.6	1	16.7	5	83.3	-
Widowed	23	10.2	3	13.0	20	87.0	-
Cohabiting	6	2.6	1	16.7	5	83.6	-
**Education level**							
No schooling	27	11.8	8	29.6	19	70.4	-
Primary	73	32.0	9	12.3	64	87.7	0.276
Junior	64	28.0	9	14.1	55	85.9	-
Senior	28	12.3	6	21.4	22	78.6	-
Tertiary	36	15.8	7	19.4	29	80.6	-
**Income level (BWP)**							
No income	84	36.8	11	13.0	73	86.9	-
Less than 1000	51	22.4	10	19.6	41	80.0	0.584
1000–2999	33	14.5	6	18.2	27	81.8	-
3000–5900	18	7.8	2	11.0	16	88.9	-
6000–9999	21	9.2	4	19.0	17	81.0	-
Above 10 000	21	9.2	6	28.6	15	71.4	-
**Duration on treatment of hypoglycaemic agents (years)**							
0–9	141	61.8	25	17.7	116	82.3	0.117
10–19	59	25.9	7	11.9	52	88.1	-
20–29	21	9.2	7	33.3	14	66.6	-
> 30	7	3.1	0	0.0	7	100.0	-
**Treatment options**							
Diet	1	0.4	0	0.0	1	100.0	-
Oral hypoglycaemic agents	113	49.6	11	9.7	102	90.3	0.012**
Insulin	35	15.4	10	28.6	25	71.4	-
Oral hypoglycaemic agents and insulin	79	34.6	18	22.8	61	77.8	-
**Family history of relative with diabete s mellitus**							
Yes	148	64.9	27	18.2	121	81.8	0.865
No	76	33.3	12	15.8	64	84.2	-
Do not know	4	1.8	0	0.0	4	100.0	-
**Most recent HbA1c**							
≤ 7	103	45.2	16	15.5	87	84.5	0.590
> 7	125	54.8	23	18.4	102	81.6	-

BWP, Botswana Pula - local currency; ITAS, Insulin Treatment Appraisal Scale; HbA1c, glycated haemoglobin; PIR, Psychological Insulin Resistance.

** , significance *p*- value < 0.05.

Overall, the mean (standard deviation [s.d.]) ITAS score for participants was 49.8 (9.5) and mean negative ITAS score was 41. Insulin naïve patients had higher ITAS score compared to insulin treated patients (54.5 vs. 45.5), reflecting lesser degree of negative attitudes towards insulin in the latter group.

Gender, income status and treatment type variables were included into the multivariable logistic regression model, *p*-value of < 0.25 as shown in Table 2. Gender (adjusted odds ratio [AOR]: 0.44; 95% CI: 0.211 – 0.921; *p* = 0.029) and type of treatment that one was currently taking at the time of data collection (AOR 1.58; 95% CI: 1.067 – 2.341; *p* = 0.023) were significantly associated with PIR (Table 2).

**TABLE 2:** Univariable and multivariable logistic regression analyses of factors associated with psychological insulin resistance among study participants.

Variables	UOR	95% CI of OR	*P*	AOR	95% CI of OR	*P*
Gender	2.500	1.237–5.052	0.011	0.441	0.211–0.921	0.029**
Income status	0.877	0.720–1.069	0.194	1.057	0.858–1.304	0.602
Treatment options	0.615	0.420–0.902	0.013	1.580	1.067–2.341	0.023**

UOR, unadjusted odds ratio; AOR, adjusted odds ratio; OR, odds ratio; CI, confidence interval.

** , significance *p*-value < 0.05.

Participants’ responses to each of the ITAS statements were analysed and grouped into five domains of PIR, namely, perceived personal blame, fear, self - pity or social stigma, perceived loss of one’s ability to control and dependence on doctor (Table 3). The most frequently expressed negative attitudes were perceived personal failure, followed by dependence on doctor as shown in Table 3.

**TABLE 3:** Participants’ responses according to psychological insulin resistance domains.

Domains of psychological insulin resistance	Participants’ responses and proportions
Perceived personal blame	48.7% believed that taking insulin means that they had failed to manage their diabetes with diet and tablets. 71.4% believed that taking insulin means their diabetes has become much worse.
Fear	38.6% of the participants were noted to have injection phobia. 55% cited fear of risk of hypoglycaemia with insulin therapy. 30.2% cited fear of weight gain with insulin use. 14.8% believed that their health will deteriorate with insulin use. 31.7% believed that insulin injections were painful.
self - pity or social stigma	34.4% believed insulin use will make other people see them as more sick. 12.7% believed injecting insulin is embarrassing. 41.8% believed that being on insulin causes family and friends to be more concerned about them.
Perceived loss of control	32.3% believed insulin makes life less flexible. 21.7% believed that insulin use takes a lot of time and energy. 21.2% believed that they would have to give up activities that they enjoy. 13.2% found to have a problem with injecting the correct amount of insulin every day. 12.7% believed that insulin use makes it more difficult to fulfil responsibilities.
Dependence	45.5% felt that insulin use was associated with more dependence on their doctor.

Three additional questions that were included to supplement questions in the ITAS scale are shown in Table 4. Of the three questions, only two differed significantly in their distribution according to PIR status *p* < 0.05 (Table 4).

**TABLE 4:** Categorical responses of patients with type 2 diabetes on statements that might be influencing their psychological insulin resistance.

Item	PIR status	Strongly disagree and disagree		Neutral		Strongly agree and agree		*P*
		*n*	%	*n*	%	*n*	%	
1. Lifestyle when on insulin therapy is expensive	PIR	47	24.9	25	13.2	117	61.9	0.001**
	NO PIR	22	56.4	4	10.3	13	33.3	
2. Healthcare workers contribute to the delay of patient starting insulin therapy	PIR	137	72.5	27	14.3	25	13.2	0.091
	NO PIR	33	84.6	2	5.1	4	10.3	
3. Healthcare workers use insulin therapy as a threat when blood glucose is not controlled	PIR	122	64.6	35	18.5	32	16.9	0.046**
	NO PIR	31	79.5	3	7.5	5	12.8	

** significance *p*-value < 0.05.

PIR, psychological insulin resistance.

## Discussion

This study aimed to determine the prevalence of PIR and associated factors among patients with type 2 diabetes mellitus attending an outpatient diabetes clinic in Botswana. Overall, over four-fifths (82.9%) of participants had PIR. The PIR in this study is comparable to that reported in a study conducted at a Kenyan hospital (82.6%) demonstrating that most of the study participants were averse to insulin therapy.^10^ Other studies conducted in similar settings of Congo and Egypt reported lower PIR prevalence of 42.7% and 40%, respectively.^11,14^

Studies performed in Asian countries (Pakistan, India, Malaysia) reported PIR prevalence of 53.29%, 51.25% and 51%,^15^ respectively. In Europe, a comparatively lower PIR prevalence of 42.5% was reported in a study among patients with poorly controlled type 2 diabetes in East London.^16^ The high prevalence of PIR in our study may be because of a weak integration of diabetes care at the primary care level, misinformation and inadequate training of patients with diabetes mellitus on disease process, progression, complications, indications and benefits of insulin therapy. Furthermore, beliefs about insulin therapy are related to culture and therefore this high prevalence could be attributed to the local (Botswana) culture.^17,18,19,20^ Also, the lack of resources (healthcare infrastructures being far away from users, the lack of healthcare workforce, the lack of glucose monitoring equipment, limited options of available pharmacological drugs, etc.) in developing countries compared to the developed countries could account for the disparity in prevalence of PIR in our setting compared to other better resourced settings.

Our results indicate that the type of treatment one was on, was associated with having PIR; adjusted odds ratio (AOR 1.58; 95% CI: 1.067–2.341). Similar findings have been reported elsewhere.^10,13,21^ Furthermore, our results indicate that PIR is particularly prevalent among insulin naïve diabetes mellitus patients in our setting. The overall ITAS score for our study participants was 49.8 with insulin naïve patients having a higher ITAS score of 54.5 than those on insulin 45.5. Both categories of patients had mean ITAS score above the 40-point cut-off for PIR. This finding is similar to that reported by Gulam et al. among Kenyan patients with type 2 diabetes.^10^

Hermanns et al. in their study reported that patients on insulin therapy and those who were initially on oral hypoglycaemic agents then later switched to insulin therapy had better appraisal of insulin therapy than those on oral hypoglycaemic agents.^22^ Although patients may have high ITAS scores before initiation of insulin therapy, when ITAS is repeated sometime after initiation of insulin therapy the ITAS score reduces.^10,22^ This suggests that negative insulin therapy appraisal may be modifiable with the initiation of insulin therapy and some barriers to uptake of insulin may be temporary. It is therefore reassuring that it is possible to improve negative insulin appraisal in settings of high PIR prevalence such as ours.

Being a male was associated with lower PIR, with male participants 0.44 times likely to have PIR compared to female participants. This is similar to results reported by other researchers.^5,15,18^ Studies have indicated that men and women have different attitudes and behaviours related to diabetes care. Males with diabetes mellitus have been observed to live more effectively with diabetes, exhibiting lesser depression and anxiety but more energy and more positive well-being when compared to their female counterparts.^23^ Traditional societal sex roles may be a barrier to female compliance with a diabetic regimen. For example, women have tasks such as taking care of the family and cooking. A woman may not be willing to change her family’s lifestyle to accommodate her health needs and may not feel that she has strong support from her family.^24^

Consistent with findings from prior studies evaluating factors associated with PIR, our results showed that age was not associated with having PIR. However, Azmiar et al. and Rita et al. reported an association between age under 50 years and PIR and cited restrictions and adaptation of lifestyle habits as having accounted for this association.^14,15^ Education level, income amount, duration of treatment with anti-hypoglycaemic agents, family history of diabetes and whether diabetes mellitus is controlled or not were not associated with PIR in our study, which is consistent with findings from a Kenyan study.^10^ However, it is worth noting that income was associated with PIR in our unadjusted model but did not reach a statistically significant level in the multivariable logistic regression model adjusted for age and treatment options.

The most frequently expressed negative attitudes domain was personal failure, which is captured by two statements: 1. believing that taking insulin meant their diabetes has become much worse -71.4%, 2. believing that taking insulin meant patient had failed to manage their diabetes with oral hypoglycaemic agents - 48.7%). These results are comparable to Gulam and colleagues’ findings of 61% and 40% in their Kenyan study.^10^ Taken together, these findings underscore the need to educate patients with diabetes about the natural history and progression of diabetes mellitus consequent to β-cell failure over time. Patients may associate insulin therapy with a sense of personal failure because of a common physician practice, where the possibility of insulin therapy is used as a threat to motivate better patient cooperation on current therapy adherence (usually oral hypoglycaemic agents and lifestyle modifications),^25^ a culture or practice that needs to change.

The next frequently expressed negative attitudes domain was dependence on the doctor, wherein 45.5% of participants in our study thought that use of insulin therapy is likely to result in patients having frequent visits to the doctor compared to those on oral hypoglycaemic agents. This is consistent with our participants perceptions that one’s use of insulin means their diabetes has worsened (71.4% of participants) and that they are seen as sicker in the community than others (34.4%).

The most cited type of fear in our study was fear of hypoglycaemia at 55% compared to needle phobia recorded by other studies. This could be because of previous bad experiences of episodes of hypoglycaemia by the participants themselves or their relatives or somebody they know in the community.^10,15,26^ Some studies have shown fear of hypoglycaemia to be mostly among young patients compared to geriatric patients.^27^ This fear of hypoglycaemia could be addressed alongside education on proper use of insulin.

Finally, of the three additional statements considered to be important for inclusion in the assessment of PIR in the local context, only two differed significantly in their distribution according to PIR status. Patients with negative attitudes towards insulin therapy had perceptions that healthcare workers do not use insulin therapy as a threat when blood glucose is not controlled and believed that lifestyle when on insulin therapy is expensive, mainly citing diet ‘diabetic diet’ as being expensive. Food insecurity has been cited in previous studies as an independent predictor to poor glycaemic control with patients with food insecurity citing difficulty following diabetic diet.^28^ More research is needed in our setting to explore strategies for assisting patients with food insecurity not only with making healthy food choices but also in accessing and preparing such foods.

### Limitations

A few limitations are worth noting when interpreting our findings. The use of convenience sampling rather than more probabilistic sampling techniques could have resulted in selective sampling bias. However, data collection spanned a period of 1 month, thus allowing for diversity of the spectrum of diabetes patients accessible during the study period.

Using an adapted tool (ITAS scale) instead of an adopted one would have been more robust in soliciting the attitudes and beliefs of Batswana on insulin therapy.

As a cross-sectional study of beliefs and attitudes the findings cannot infer any causality nor predict actual insulin use behaviours.

The ITAS instrument by its design does not cover aspects of the healthcare provider-related causes of PIR or coexisting emotional distress or depression. Adopting other instruments would have been helpful to adjust for these confounding influences.

## Conclusion

Prevalence of PIR in our study was high at 82.9%, mainly driven by patient and health system related factors. The high PIR may contribute to poor diabetes control and poor treatment outcomes in this setting. Knowledge of these factors may help healthcare workers in tailoring their pre-counselling sessions before initiation of insulin therapy. Personal blame was one of the negative attitudes that was prevalent in our study; this indicates a need for enhanced patient education on the progressive nature of the disease.

Further studies evaluating the influence of healthcare workers on PIR among patients with diabetes mellitus are important, as healthcare workers have been implicated in delays to initiating insulin therapy on patients (clinical inertia).
